# Detection of scorpion venom by optical circular dichroism method

**DOI:** 10.1038/s41598-021-95493-7

**Published:** 2021-08-04

**Authors:** Y. Mazhdi, S. M. Hamidi

**Affiliations:** Magneto-Plasmonic Lab, Laser and Plasma Research Institute, Shahid Beheshti University, Tehran, Iran

**Keywords:** Optical physics, Optical techniques, Circular dichroism

## Abstract

Various efforts have been made to detect minimum amounts of any toxic materials in water or the neurotoxic effect of venom (*Odontobuthus Doriae* Scorpion) in the human’s blood serum nerve by high-sensitivity, accurate, and low-cost sensors in order to enhance life style. Therefore, the present study was done to investigate reliability of two-dimensional plasmonic structure and circular dichroism (CD) in toxic samples in order to measure and determine venom concentrations and its neurotoxic effect on humans҆ blood serum Neurotransmitter analytes. Our results confirmed dependency of CD signal to neurotoxic effect of venom concentrations and good sensitivity of this sensor with the help of achiral plasmonic structure.

## Introduction

Chirality as a useful and key aspect in molecular biology can be used for biosensing applications^1^. Given importance of high-resolution and low-cost biosensors, many studies have been conducted to enhance chirality through the use of new materials and chiral and achiral nanostructures^2^. A wide range of these nanostructures consist of symmetry breaking ones like chiral oligomers^3^, chiral nanoparticles^4^, nanohelix arrays^5^, twisted nanorods^6^, three-dimensional helices^7^; or symmetric ones like square array of nanoparticle platform^8^ that are used to enhance circular dichroism(CD) signal. It is well known that the absence of mirror symmetry or quasi two- or three-dimensional thin films lacking plane mirror symmetry is the main key factor in achieving the above-mentioned chirality^9^. Currently, experimental and theoretical evidence shows that surface plasmon polariton (SPP) waves in achiral structures can show chirality due to asymmetry in field distribution^10,11^.

Scorpion venom causes various complications including local pain, inflammation, necrosis, and blood-related effects and envenomation affects nervous system quickly and within a few hours^12^. It causes temporal paralysis, involving a wide range of injuries, from mild to moderate and severe, affecting one muscle group or the whole body^13^. Considering these warning and dangerous effects of this venom, recognizing its cholinergic and neurological effects on humans online and in the short time, is of great importance in very low concentrations. So far, many chemical methods have been used to detect and distinguish this kind of venom in different concentrations; but determination of the minimum amounts by invasive methods like optical ones is so important now. Among optical methods, there is an optical sensor based on surface plasmon resonance (SPR) that is very famous and is used to determine the minimum concentrations of any venoms with higher sensitivity based on online changes in metal refractive index and dielectric adjacent environment.

*Odontobuthus doriae* scorpion is one of the most dangerous species of scorpion in Iran, venom of which was detected its neurotoxic effect on humans҆ blood serum in this study using the SPR sensor designed and fabricated based on the above-mentioned chirality in metallic substrate.

## Experimental setup

Fabrication of 2D grating using the soft imprint lithography method can offer some advantages, such as reasonable cost, high efficiency, and reproducible manufacturing at large scale, so here, this method was used for fabrication of 2D grating^14^. For this purpose, unique codes and corresponding resolution were extracted using a 2D charge-coupled device (CCD) of a camera. Then, this CCD was attached to a glass substrate by a two-sided adhesive tape. Square unit cell of the CCD had the periodicity of 3.10 μm. After mixing polydimethylsiloxane (PDMS) and curing agent (1:10) for 5 min, the mixture was poured on the template with an approximate thickness of 1.4 mm. For fabricating our 2D plasmonic structures, the SYLGARD 184 elastomer kit provided by Sigma-Aldrich Company was used. For eliminating the probable bubbles from the mixture, the sample was inserted in a rotary vacuum pump for 15 min and then, the samples were put on a heater for 30 min at 50 °C, 15 min at 75 °C, and 15 min at 100 °C, respectively. After one day, the PDMS samples were peeled off from their molds. At the next step, surfaces of the samples were approximately coated with about 35 nm of gold by a physical vapor deposition machine.

**Figure 1:**
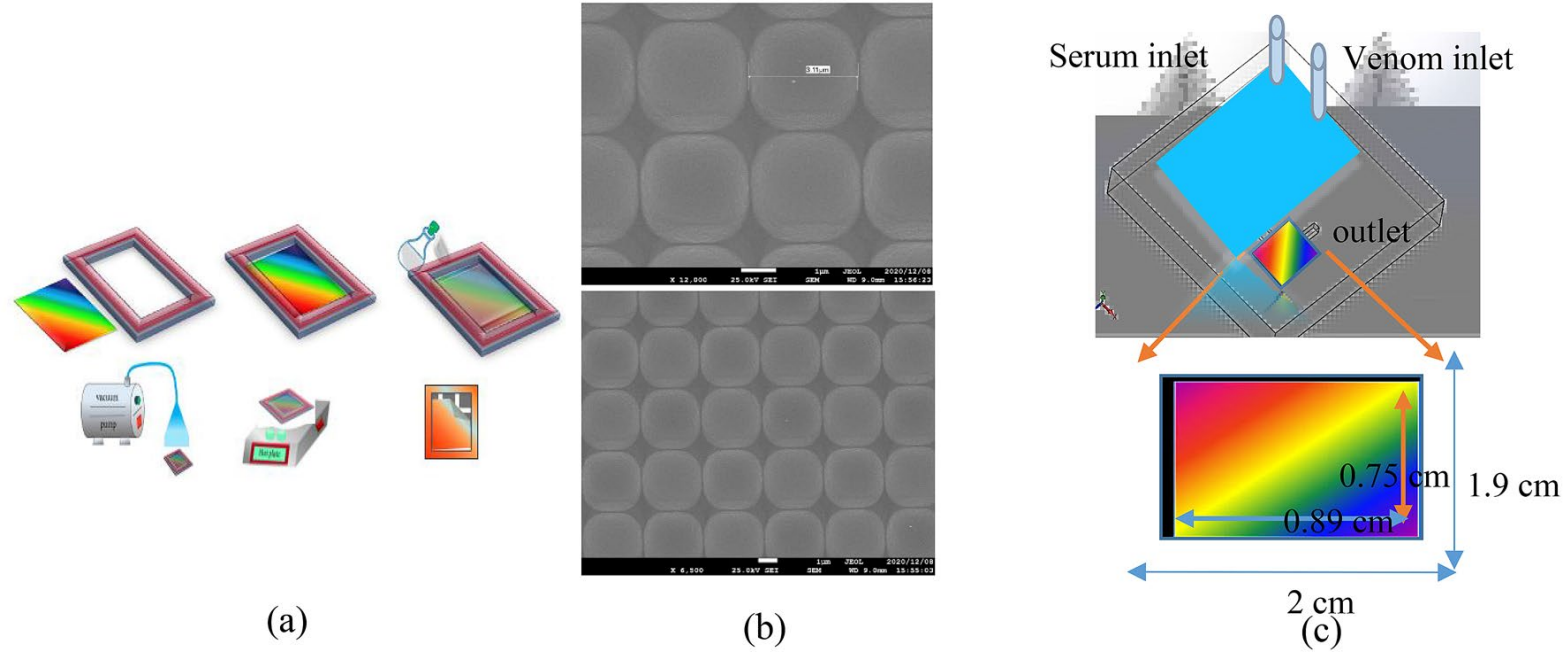
(**a**) schematic diagram of soft nanolithography, (**b**) SEM graph of samples and (**c**) main channel without (top) and with (bottom) plasmonic sensor.

Figure 1a schematically shows fabrication process of a 2D plasmonic grating. Accordingly, the patterned PDMS as a grating was observed with periodicity of 3.11 μm in scanning electron microscopy (SEM) image taken from the control sample as presented in Fig. 1b. In addition, schematic of the 2D regular structure covered with gold is also shown in Fig. 1b. Actually, this structure was selected since 2D grating acts like a Bragg reflector due to its bandgap in reflection spectrum in visible regim ^15,16^ as well as providing a good surface lattice resonance because of gold nanorods at the interface of each unit cell. For investigating the effect of scorpion venom on stimulation of nervous system in humans҆ neurons in transmission measurement setup, fluidic channel was required according to Fig. 1c. For this purpose, a transparent flow cell with two inlet channels of the same size (top image) was designed for simultaneous and equal entry of humans҆ serum and different concentrations of scorpion venom using laser incisions on a transparent plexiglas sheet with a thickness of 2 mm. A circular cavity of 2 mm thickness was embedded inside the flow cell and in passage of blood serum and scorpion venom solution to combine the two inputs in the flow cell. Blood serum was collected from laboratory with External Quality Assurance Services (EQAS) with identification number 2740. The blood sample was taken as a blood clot in a gel tube for 20 min at ambient temperature and then centrifuged at 2500 rpm for 10 min, the serum was removed and poured into a falcon.

In addition, the generated sensor chip was attached to the cube embedded inside the flow cell so that, its gold-coated surface was adjacent to the material passing through the flow cell (bottom image).

For testing sensor's capability and sensitivity to venom, three concentrations of 5, 20, and 40 ppm were needed, which were prepared as follows: first, 2 mg of scorpion venom was dissolved, as a white powder in 1 ml of phosphate-buffered saline (PBS) biological solution. After 10 min of centrifugation, 0.05, 0.2, and 0.4 mg of the solution were dissolved in three equal amounts of 20 ml of PBS to prepare concentrations of 5, 20, and 40 ppm as S_1_, S_2_ and S_3_ samples, respectively.

Moreover, for preparing 50 ml of healthy humans҆ blood serum, 150 ml of blood was collected and after clotting, it was centrifuged and then, the serum was separated. These prepared samples were studied in CD experimental setup, in which sample's transmission was documented after excitation by right and left circularly polarized (RCP and LCP) light and difference between them was recorded in the visible region.

### Sample guideline

All methods were carried out in accordance with relevant guidelines and regulations in Noor Pathobiology and Genetics Laboratory with registration number 30859/4/26/P by the Ministry of Health and Medical Education of Iran has a certificate of participation in External Quality Assurance Services (EQAS) with identification number 2740.

### Human sample guideline

This study was approved by the Ethics Committee of the "Ethical committee of Vice president of research of Shahid Beheshti university/IR.SBU.REC.1400.

## Results and discussions

**Figure 2:**
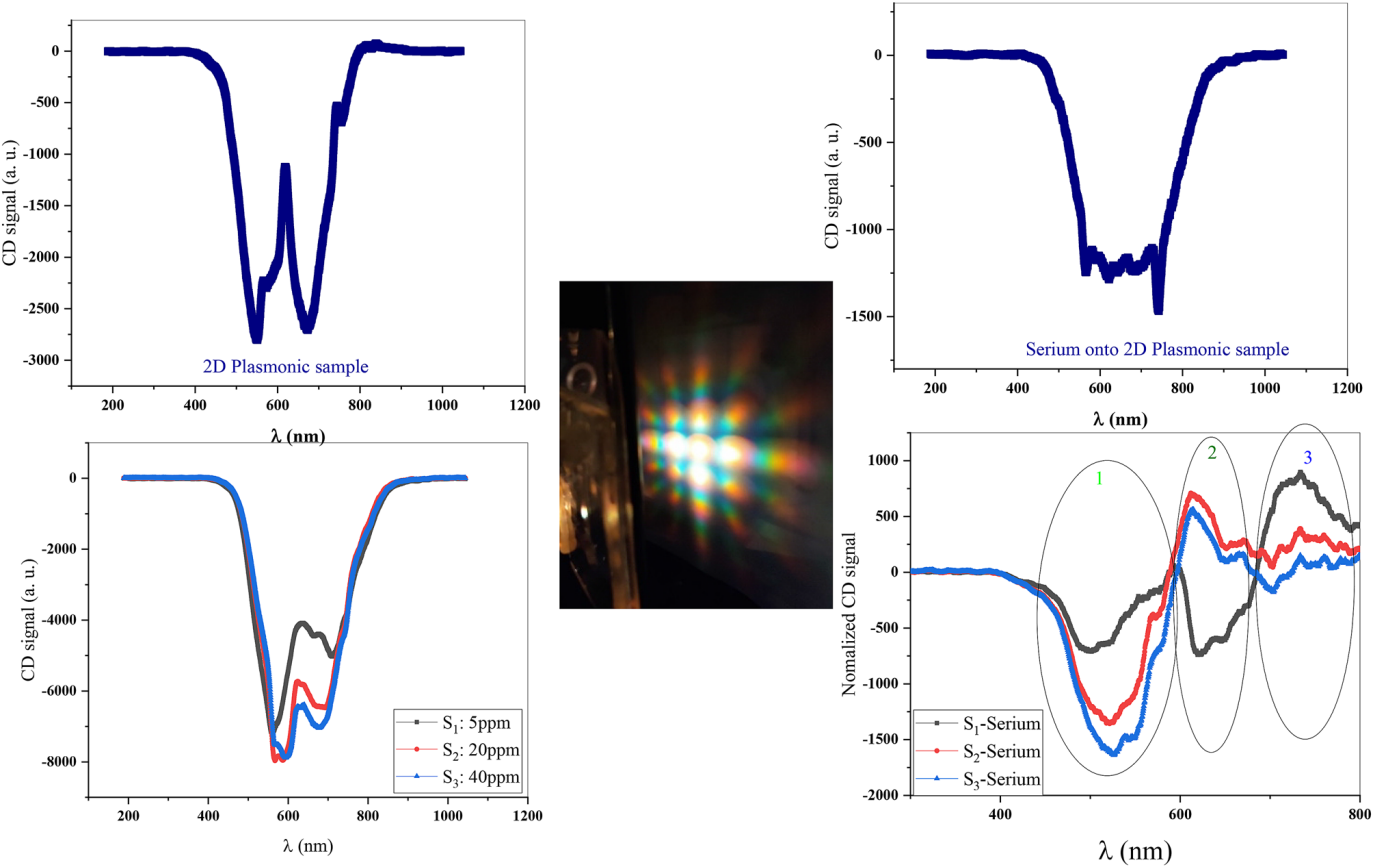
CD signal of (**a**) bare 2D plasmonic sample, (**b**) 2D plasmonic sample as serium sensor, (**c**) 2D plasmonic sample as Venom senor in three different concentrations and (**d**) normalized CD signal or three different venom concentrations.

CD spectra of the sample as difference in transmission spectra between LCP and RCP light are shown in Fig. 2. Figure 2 shows CD spectra for two-dimensional (2D) bare sample, serum placed onto 2D plasmonic sample, three different S_1_–S_3_ samples and also, normalized CD signal as a difference between CD signals in the presence of venom concentrations and serum sample in (a), (b), (c), and (d) sections, respectively.

Observed chirality due to achiral structure and tilt with respect to normal sample's surface appeared by asymmetric field distribution pattern as a result of asymmetric plasmon modes excited by RCP and LCP light. These interesting features can result from nanowires in each motif of plasmonic square array. In fact, in each angle, a percentage of wires' projection and thus, plasmonic field yield changes in transmission consequently, leading to chiro-optical effects.

As shown in Fig. 2a, there is overlap between the localized surface plasmon resonances (LSPR) due to nanowires and diffraction orders by lattice yielding to surface lattice resonance (SLR) in CD signal resonance as explained in our previous reports^17^. This can be attributed to the fact that transmission spectrum of the sample shows two resonance dips at 550 and 673 nm because of SLR and extraordinary optical transmission (EOT) of nano plasmonic structure.

Importantly, the signal appears near SLR and when unknown sample is placed on surface of sensor, which can be sensed through any change in CD signal. Furthermore, sign of chirality is reversed after passing from SLR wavelength that is contrary to the fact that exactly in resonance region, trend of enhanced chirality was stopped due to the increase in venom concentration that the main and dominant effect of this increase in concentration is directly on neurotoxicity, as a result of changing the concentration of the main neurotransmitter analytes in the blood serum. According to research, this complication is the predominant cause of many diseases caused by scorpion bites^13^.

As mentioned in the introduction, scorpion venom, due to being a neurotoxin, has many effects on the nervous system at the presynaptic level (β-neurotoxin) and postsynaptic level (α-neurotoxin)^12^, which β-neurotoxin inhibit the release of acetylcholine or noradrenaline which are neurotransmitters and α-neurotoxin reversibly blocks acetylcholine receptors at the postsynaptic level^13^. The main reason for the effect of scorpion venom on the central and peripheral nervous system as well as irritable tissues in muscles is the ability of this toxin to interact with Na^+^, K^+^, Ca^2+^, and Cl^−^ channels, which causes metabolic impacts^18^. We used Iranian yellow scorpion venom in this study, which is a species of *doriae* in the *family Buthidae*. The *O.doriae* venom is able to interact with some of voltage-dependent channels of sodium (V_Na_) and potassium (V_K_). According to a study conducted in 2003 (at Shahid Sadoughi University of Medical Sciences in Yazd City^21^). In this way, after injecting scorpion venom into the living organism, its blood serum is separated and at different times, the amount of total protein, total bilirubin, uric acid, cholesterol, amylase enzyme and electrolytes (Cl^−^, K^+^, Na^+^) are analyzed. Evaluation of statistical results of serum biochemical parameters shows that the electrolytes (Cl^−^, K^+^, Na^+^) in the tested samples compared to the control group, at a time between 5 to 15 min after injection, had a significant decrease (P < 0.05)^22^. This decrease is due to cholinergic effects and vomiting. Also, no significant difference was observed in other biochemical parameters. Therefore, it can be said that in poisoning with *Odontobuthus doriae* scorpion venom, the first cholinergic effects of the venom occur and it is completely dominant over the other effects. Therefore, scorpion venom has a direct effect on the amount of serum electrolytes. It reduces amount of electrolytes and because of neuronal signal of the humans҆ central nervous system causes changes in concentration of these electrolytes. When these electrolytes in an analyte are placed near metal surface of the plasmon sensor, they form a layer near metal surface with an action potential of V_0_, which is known as Stern layer. This created neuronal activity and action potential that causes physical changes is the basis of our sensory approach. This action potential is unique to each specific neurotoxin and its different injected concentrations. In other words, any neurotoxin that binds to tissues or human neurotransmitters creates its own unique action potential, which in our sensing method determines the type and concentration of the toxin. Therefore, with changes in electrolytes, the potential size of the Stern layer changes and according to the Drude–Stern relation, refractive index of the metal also changes so that, resonant frequency of surface plasmons varies and reveals the neurological effects of toxin on the humans҆ central nervous system^19^.

1 $${\Delta \lambda }_{LSP}=-\frac{{\epsilon }_{0}{\omega }_{P}^{{*}^{2}}{\lambda }_{LSP}^{3}}{8{\pi }^{2}{C}^{2}{Nedd}_{TF}({\epsilon }_{\infty }+\frac{1-L}{L})}{V}_{0}$$

where, ${\epsilon }_{0}is$ the electric permittivity of vacuum and d is the distance between the plates (the plasmonic template is assumed to be at ground potential). d_TF_ is the Thomas–Fermi screening length and N and e are electron number density and elementary charge, respectively, ω_p_* is the gold plasma frequency, and static dielectric constant ε_∞_ accounts for background polarization because of the presence of core electrons. λ_LSP_ is the resonance wavelength of LSP and c is the speed of light in vacuum. L is the geometrical factor in polarization direction of incident electromagnetic wave, and V_0_ is an applied voltage.

Our simulation results approved that CD takes place in the same resonance wavelength as SLR of the sample for normal incidence and 5-degree tilt angle, confirming the main role of SPR in this finding.

**Figure 3:**
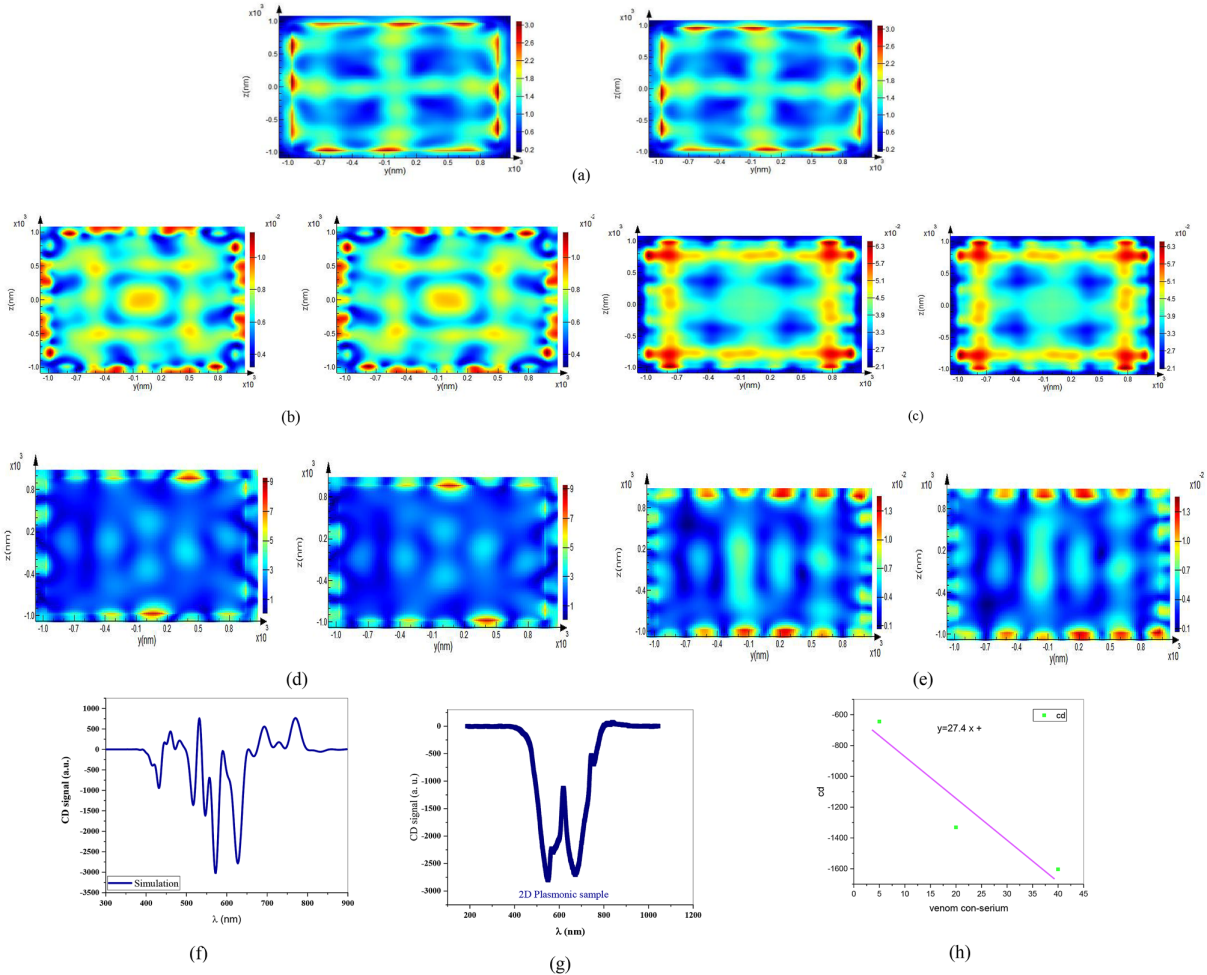
Magnetic and Electric field distributions of sample for right and left circular polarizations for (**a**) LCP-T-E and RCP-T-E and (**b**) LCP-T-H and RCP-T-H and (**c**) LCP-R-H and RCP-R-H and (**d**) LCP-T-E and RCP-T-E for normal incidence and (**e**) LCP-T-H and RCP-T-H for 5-degree incidence angle. (**f**) Simulation of CD signal, (**g**) measured CD signal in normal incidence and (**h**) sensitivity diagram of the sensor.

Symmetry breaking in tilt incidence angle is very stronger due to SPR in corners of each motif. Besides, it approves enhancement factor of signal in incidence angle, which is a bit more than normal ones, for example by 5 degrees. This fact is the result of non-symmetric SPR excitation in each nanowire in corners of motifs as shown in Fig. 3.

In our 2D plasmonic sample with perfect mirror symmetry in tilt angle, while vector of incident radiation wave was in the sample plane by any of the unit cell lattice and formed a triangle with two structure lattice constants, SPR appeared as a result of plasmonic dipole excitation. In direction of the radiation wave vector in the sample plane, known as direction of dipole screw, this total wave vector is the result of constructive interference of the individual SPPs of wires with each other, showing dependence of this final wave vector on phase of each single SPP wave vector.

Consequently, asymmetry near field created by this SPP wave causes plane mirror symmetry of the array structure to be broken and makes this structure to have an optical chirality effect.

Since, the SPP vector of array in metal includes both real and imaginary parts; this structure is expected to produce a significant CD signal due to the presence of imaginary part of the wave vector. Obviously, the interfering effect of SPP of each hole at metal-dielectric boundary is directly dependent on properties of both environments, so change in dielectric environment alters phase matching conditions of the SPP interference and produces a different CD signal as explained in the literature^20^.

As can be seen in the diagram, the amplitude of the circular dichroism signal is amplified by increasing different concentrations of scorpion venom in the blood serum, which is a sign of the sensitivity of the proposed sensor to nerve stimulation by scorpion venom in the blood serum. The values of the CD signal at the 520 nm wavelength, which is approximately the same as the SLR wavelength and near the initial wavelength of the EOT wavelength rang, are plotted for three different concentrations of venom in the blood serum to get the sensitivity of S = 27.4 as shown in Fig. 3h.

As discussed, the results clearly show that any change that affects the amount of neurotoxicity of venom, whether changes in the type of toxin or changes in its concentration, will affect the sensor response because any change in the toxin will cause a change in the action potential, which depends on the type and concentration of the toxin. Therefore, this approach, which is done completely online, is sensitive to any changes in the toxin and only delay in the measurement is related to the time of effect of the toxin on serum analytes, which lasts between 5 and 15 min.

## Conclusion

In conclusion, achiral plasmonic structure was used in this study as highly sensitive and low-cost sensor to detect neuronal activity of venom of the *Iranian Odontobuthus doriae* scorpion in humans҆ blood serum. Our results showed that changes in venom concentrations caused alterations in amount of electrolytes and thus, action potential of the Stern layer and according to the Drude–Stern relation, refractive index of the metal changed in our 2D plasmonic substrate. Resonant frequency of SLR in the main sample was altered by the neurological effect of the toxin on the humans҆ central nervous system and thus, it can be said that any change in venom concentration can be sensed. Finally, in this achiral sensors, resonance wavelength and thus CD signal has been affected by any change in the lattice plasmon polaritons by creating action potential at the sample surface. This action potential is unique to each specific neurotoxin and its different injected concentrations. In other words, any neurotoxin that binds to tissues or human neurotransmitters creates its own unique action potential, which in our sensing method determines the type and concentration of the toxin.

## References

[1] Hendry E, Carpy T, Johnston J, Popland M, Mikhaylovskiy RV, Lapthorn AJ, Kelly SM, Barron LD, Gadegaard N, Kadodwala M (2010). Ultrasensitive detection and characterization of biomolecules using superchiral fluids. Nat. Nanotechnol..

[2] Gordon R, Brolo AG, McKinnon A, Rajora A, Leathem B, Kavanagh KL (2004). Strong polarization in the optical transmission through elliptical nanohole arrays. Phys. Rev. Lett..

[3] Le KQ (2018). Enhanced circular dichroism via symmetry breaking in a chiral plasmonic nanoparticle oligomer. J. Electron. Mater..

[4] Petronijevic E, Ali H, Zaric N, Belardini A, Leahu G, Cesca T, Mattei G, Andreani LC, Sibilia C (2020). Chiral effects in low-cost plasmonic arrays of elliptic nanoholes. Opt. Quant. Electron..

[5] Gibbs JG, Mark AG, Eslami S, Fischer P (2013). Plasmonic nanohelix metamaterials with tailorable giant circular dichroism. Appl. Phys. Lett..

[6] Zhao S-X, Zhang W (2019). Plasmonic chirality of one-dimensional arrays of twisted nanorod dimers: The cooperation of local structure and collective effect. Opt. Express.

[7] Cao T, Wei C, Mao L, Li Y (2014). Extrinsic 2D chirality: Giant circular conversion dichroism from a metal-dielectric-metal square array. Sci. Rep..

[8] Mohammadi E, Tsakmakidis KL, Askarpour AN, Dehkhoda P, Tavakoli A, Altug H (2018). Nanophotonic platforms for enhanced chiral sensing. ACS Photon..

[9] Wang X, Tang Z (2017). Circular dichroism studies on plasmonic nanostructures. Small.

[10] Horrer A, Zhang Y, Gerard D, Beal J, Kociak M, Plain J, Bachelot R (2020). Local optical chirality induced by near-field mode interference in achiral plasmonic metamolecules. Nano Lett..

[11] Maoz BM, Moshe AB, Vestler D, Bar-Elli O, Markovich G (2012). Chiroptical effects in planar achiral plasmonic oriented nanohole arrays. Nano Lett..

[12] Jalali A, Bosmans F, Amininasab M, Clynen E, Cuypers E, Zaremirakabadi A, Sarbolouki M-N, Schoofs L, Vatanpour H, Tytgat J (2005). OD1, the first toxin isolated from the venom of the scorpion *Odonthobuthus doriae* active on voltage-gated Na+ channels. FEBS Lett..

[13] Vatanpour H, Jalali A, Rowan EG, Rahim F (2013). Effects of odontobuthus doriae scorpion venom on mouse sciatic nerve. IJPR.

[14] Saeidifard S, Sohrabi F, Ghazimoradi MH, Hamidi SM, Farivar S, Ansari MA (2019). Two-dimensional plasmonic biosensing platform: Cellular activity detection under laser stimulation. J. Appl. Phys..

[15] Asgari N, Hamidi SM (2018). Exciton-plasmon coupling in two-dimensional plexitonic nano grating. Opt. Mater..

[16] Sohrabi F, Etezadi D, Perin R, Jahani Y, Mohammadi E, Hamidi SM (2020). Phase-sensitive optical neural recording of cerebellum tissue on a flexible interface. J. Appl. Phys..

[17] Mbarak H, Ghahrizjani RT, Hamidi SM, Mohajerani E, Zaatar Y (2020). Reversible and tunable photochemical switch based on plasmonic structure. Sci. Rep..

[18] Maertens C, Cuypers E, Amininasab M, Jalali A, Vatanpour H, Tytgat J (2006). Potent modulation of the voltage-gated sodium channel Nav1.7 by OD1, a toxin from the scorpion *Odonthobuthus doriae*. Mol. Pharmacol..

[19] Zhang J, Atay T, Nurmikko AV (2009). Optical detection of brain cell activity using plasmonic gold nanoparticles. Nano Lett..

[20] Wang X, Pang Z, Tong H, Wu X, Bai X, Yang H, Wen X, Qi Y (2019). Theoretical investigation of subwavelength structure fabrication based on multi-exposure surface plasmon interference lithography. Results Phys..

[21] Jafari SS, Khamesipour VR (2001). The effects of different doses of scorpion (Odontobuthus) venom on some changes of biochemical factors of blood serum in dogs. Daneshvar.

[22] Ismail M (1994). The treatment of the scorpion envenoming syndrom. Toxicon.

